# Insight into Microevolution of *Yersinia pestis* by Clustered Regularly Interspaced Short Palindromic Repeats

**DOI:** 10.1371/journal.pone.0002652

**Published:** 2008-07-09

**Authors:** Yujun Cui, Yanjun Li, Olivier Gorgé, Mikhail E. Platonov, Yanfeng Yan, Zhaobiao Guo, Christine Pourcel, Svetlana V. Dentovskaya, Sergey V. Balakhonov, Xiaoyi Wang, Yajun Song, Andrey P. Anisimov, Gilles Vergnaud, Ruifu Yang

**Affiliations:** 1 State Key laboratory of Pathogen and Biosecurity, Institute of Microbiology and Epidemiology, Beijing, China; 2 Univ. Paris-Sud 11, CNRS, UMR8621, Institut de Génétique et Microbiologie, Orsay, France; 3 State Research Center for Applied Microbiology, Obolensk, Moscow Region, Russia; 4 Antiplague Research Institute of Siberia and Far East, Irkutsk, Russia; 5 DGA/D4S-Mission pour la Recherche et l'Innovation Scientifique, Bagneux, France; Centre for DNA Fingerprinting and Diagnostics, India

## Abstract

**Background:**

*Yersinia pestis*, the pathogen of plague, has greatly influenced human history on a global scale. Clustered Regularly Interspaced Short Palindromic Repeat (CRISPR), an element participating in immunity against phages' invasion, is composed of short repeated sequences separated by unique spacers and provides the basis of the spoligotyping technology. In the present research, three CRISPR loci were analyzed in 125 strains of *Y. pestis* from 26 natural plague foci of China, the former Soviet Union and Mongolia were analyzed, for validating CRISPR-based genotyping method and better understanding adaptive microevolution of *Y. pestis*.

**Methodology/Principal Findings:**

Using PCR amplification, sequencing and online data processing, a high degree of genetic diversity was revealed in all three CRISPR elements. The distribution of spacers and their arrays in *Y. pestis* strains is strongly region and focus-specific, allowing the construction of a hypothetic evolutionary model of *Y. pestis*. This model suggests transmission route of microtus strains that encircled Takla Makan Desert and ZhunGer Basin. Starting from Tadjikistan, one branch passed through the Kunlun Mountains, and moved to the Qinghai-Tibet Plateau. Another branch went north via the Pamirs Plateau, the Tianshan Mountains, the Altai Mountains and the Inner Mongolian Plateau. Other *Y. pestis* lineages might be originated from certain areas along those routes.

**Conclusions/significance:**

CRISPR can provide important information for genotyping and evolutionary research of bacteria, which will help to trace the source of outbreaks. The resulting data will make possible the development of very low cost and high-resolution assays for the systematic typing of any new isolate.

## Introduction

*Yersinia pestis*, the causative agent of plague, is a Gram-negative bacterium that belongs to *Enterobacteriaceae*. Four biovars (bv.), including antiqua, orientalis, medievalis and microtus, are defined by biochemical characteristics and the first three are significantly pathogenic for humans. In the recorded history, three waves of human plague pandemics have led to the death of millions of people [1] and resulted in major social changes. The main reservoir for *Y. pestis* is rodents and vector insects (usually fleas). Until now, *Y. pestis* has been found in more than 200 species of wild rodents inhabiting in plague foci in all the continents except Australia and Antarctica [2], [3]. Because of its characteristics, *Y. pestis* is included in the selected list of the bioterrorism-related agents [4]–[6].

Although natural plague foci are widely dispersed in the world, most of this geographic spread is the result of the third pandemic starting in the mid-19^th^ century from the Yunnan province of China. Accordingly, the diversity of the strains found in the recent foci, including North and South America, is very limited within or across the foci [7], [8]. In contrast, the distinct foci in regions of Central and East Asia, especially China, the former Soviet Union (FSU) and Mongolia, harbor diverse strains, which can be estimated from both biochemical data and host diversities. Human plague has been well controlled in China since the 1950s, but at least 12 types of natural plague foci still exist, covering 241 counties in 15 provinces. Beside the classical bv. typing, Chinese isolates of *Y. pestis* have been divided into 18 ecotypes, based upon several biochemical features, including glycerol, rhamnose, maltose, melibiose, and arabinose fermentation, nitrate reduction, amino acid utilization, mutation rate from Pgm^+^ to Pgm^−^, and water soluble protein patterns on SDS-PAGE [9]. Based on results of extensive microarray and PCR analysis, 32 genomovars, including 14 major genomovars and 18 minor genomovars, were identified for Chinese strains according to different region (DFR) profiles [10], [11]. Similarly, different types of foci have been identified in the FSU (including the foci in Russia, Kazakhstan, Georgia, Armenia, Azerbaijan, Turkmenistan, Uzbekistan, Tadjikistan and Kirghizia ) and Mongolia, and Russian scientists have classified isolates from these foci into different subspecies by various biochemical and molecular biological methods [3]. The significant diversity revealed by both genotyping and phenotyping among *Y. pestis* isolates from above natural plague foci suggests that further insights into genetic diversity of plague bacteria isolated from these regions will help better understand molecular microevolution of *Y. pestis*.

Several molecular methods have been used for typing *Y. pestis*, with variable clustering and discriminating ability. Molecular methods like ribotyping and multilocus sequence typing (MLST), as well as restriction fragment length polymorphism (RFLP), pulsed-field gel electrophoresis (PFGE), and randomly amplified polymorphism DNA (RAPD) etc., have no or very low discrimination power [12]–[15]. Conversely, insertion sequence (IS) typing by Southern blotting reveals a huge diversity, but it does not provide an appropriate phylogenetic or clustering tool. Furthermore, patterns obtained in different laboratories are difficult to compare. Multiple Locus VNTR (Variable Number of Tandem Repeats) analysis (MLVA), which usually provides a good differentiation of isolates [16], has been developed essentially by two groups for typing *Y. pestis* [8], [17]. Similarly, Single nucleotide polymorphism (SNP) analysis will offer an overview of *Y. pestis* microevolution, shaping the different evolutionary branches from its ancestor *Yersinia pseudotuberculosis* [7], [13] as the genomic sequences from a growing number of representative strains for each subspecies and clade will be available.

Previous work [18] suggested that the investigation of Clustered Regularly Interspaced Short Palindromic Repeats (CRISPRs) could provide some clues to the evolution of *Y. pestis*, and predictions were made concerning the CRISPR organization of ancestral *Y. pestis* strains [19]. CRISPRs are a family of elements which typically consist of noncontiguous direct repeats (DR, 24 bp–47 bp) separated by stretches of similarly sized unique sequences [20], [21]. One or more CRISPR loci are found in 40% of the bacterial genomes sequenced so far and in most archaea [22]. CRISPR loci, Cas (CRISPR-associated) proteins, and leader sequences (the non-coding sequences flanking the CRISPR loci on one side and acting as a promoter) [21], were suggested to constitute a prokaryotic immune system against bacteriophage attack. This was demonstrated in *Streptococcus thermophilus* [23]–[25].

The unique sequences in CRISPR loci, “spacers”, show a general divergence within a given species. A fraction of spacers are observed to be homologous with preexisting sequences such as bacteriophage and conjugative plasmids [18], [26]–[28]. Diversity within a CRISPR locus was first used for typing *Mycobacterium tuberculosis* strains. The assay called spoligotyping (spacer oligonucleotide typing) screens 43 spacers by hybridization using a nylon membrane[29]. An international database, comprising more than 2, 000 patterns from almost 40, 000 isolates, is accessible through the internet [30]. More recently, a similar assay was applied for genotyping *Corynebacterium diphteriae* [31].

Previous investigations were done on *Y. pestis* strain collections representing only a limited genetic diversity of the species. In particular, strains associated with the third pandemic were largely overrepresented. In the present work, we explore the potential use of the CRISPR loci in genotyping and evolutionary research of *Y. pestis*. In order to define a representative strain collection, polymorphic tandem repeat analysis was applied to the typing of more than 400 new *Y. pestis* isolates collected from 12 natural foci of China, 12 natural foci of the FSU, and 2 natural foci of Mongolia, including strains from the three classical biovars and bv. microtus (Pestoides group or non-*pestis* subspecies). A representative collection of 125 strains (Table S1) could be defined within which the CRISPR loci were characterized by sequencing in order to identify spacers for microevolution analysis and future development of typing assays.

Three hundred and sixty-four CRISPR alleles were sequenced and 86 new spacers were identified. We found that various spacers/spacers arrays had obvious connection with geographic source, and an evolutionary model of *Y. pestis* was proposed. Additional insights into common characteristics of CRISPR elements were obtained by integrating CRISPR data from previous research [18], [19].

## Results and Discussion

### CRISPR loci in the seven sequenced *Y. pestis* genomes

**Figure 1:**
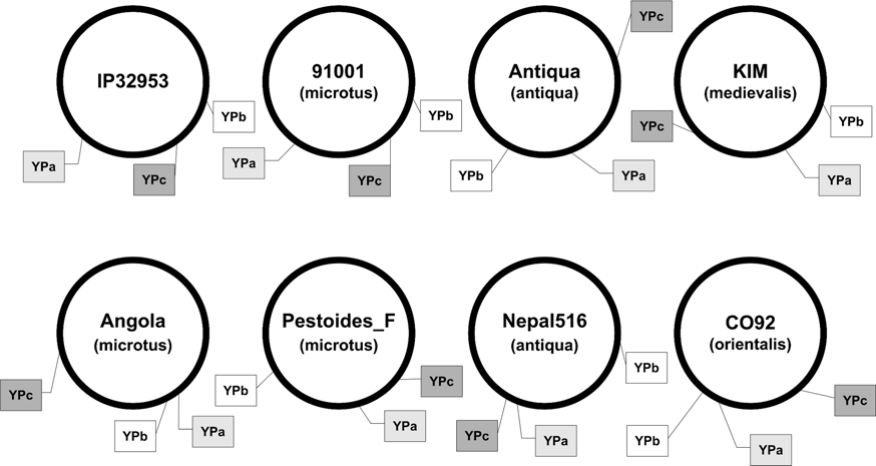
The relative position of CRISPR loci in the sequenced genomes of seven *Y. pestis* and one *Y. pseudotuberculosis* (IP32953).

Each of the seven sequenced *Y. pestis* genomes contains three CRISPR loci (YPa, YPb and YPc, which were referred to YP1, YP2 and YP3, respectively, in a previous report [18]). They are localized on different positions in the sequenced genomes due to DNA rearrangements (Figure 1). The DRs are conserved within these three loci with a sequence of 5′- TTTCTAAGCTGCCTGTGCGGCAGTGAAC-3′, and there is a truncated DRs in the 5′ end of each CRISPR locus with the sequences of 5′- TGCCTGTGCGGCAGTGAAC -3′, 5′- TAAGCTGCCTGTGCGGCAGTGAAC -3′ and 5′- GCTGCCTGTGCGGCAGTGAAC -3′ for YPa, YPb and YPc, respectively. The DR sequences (including the first truncated DR) are identical in all the strains analyzed, suggesting that the conserved repeat sequences are important for *Y. pestis*. Interestingly, in YPb and YPc of the Angola strain, there is only a truncated DR and a leader sequence. There are 6 *cas* genes in *Y. pestis* genomes (YPO2462∼2465, YPO2467 and YPO2468 in the CO92 genome, all of them belong to the Ypest subtype defined by Haft *et. al.* [32]), localized in the flanking region of YPa. According to the model proposed by Grissa *et al.* [22], YPb and YPc would represent secondary loci produced from an initial locus (YPa) containing and providing all the necessary machinery. The leader sequences are similar in YPa and YPb, but less conserved in YPc.

### Spacers sequences in *Y. pestis*

Including the 45 spacers previously identified [18], a total of 131 spacers have been found in *Y. pestis* until now (Table S2). The average GC content of the spacers is 47.2%, a little bit lower than that of *Y. pestis* genomes (47.7%). Herein 83, 37 and 11 spacers were found for YPa, YPb and YPc, respectively, with a length ranging from 29–34 bp, mostly 32 bp (79%).

Seventy seven (59%) of the 131 spacers have homologous sequences to the proto-spacers [23], [24] in a prophage (YPO2096∼2135 in CO92 genome), whereas 22 spacers (17%) are homologous to other non-viral regions in the *Y. pestis* chromosome (Table S3). No significant similarity was found in the public sequence databases for the remaining 32 spacers. It has been demonstrated recently [33] that a prophage (Ypf*Φ*, YPO2271∼YPO2281 in CO92 genome) was stabilized in the orientalis strains, and was present unstably in some isolates of the other three *Y. pestis* biovars as an episome [11]. The interaction between Ypf*Φ* and *Y. pestis* could have left some trails in the CRISPR loci, however, none of the identified spacers in *Y. pestis* have an homologue in Ypf*Φ*. Three genes in the prophage region, YPO2106, YPO2108 and YPO2116, contain 15, 15 and 10 proto-spacers respectively, whereas the other genes in this region have less than five (Figure S1). Those three genes seem to be hot spots for providing spacers, suggesting that they might play important roles during the fighting against phages. Interestingly, the proto-spacer for spacer b29 (subsequently called p-b29) is located in IS*285* (21 copies in the CO92 genome), and p-a30 is in 23S rRNA (6 copies in the CO92 genome). The number of proto-spacers originating from the *Y. pestis* genome itself is remarkably higher than that from other species, including its very close neighbor *Y. pseudotuberculosis*, suggesting that the CRISPR loci in *Y. pestis* might play a role in terms of shaping the genome expression, in addition to acting as a defense mechanism against phage infections. As previously observed, proto-spacers were present in both strands of the corresponding genes in the genome (Table S2). This could indicate that the two strands of the spacers are eventually produced as small interfering RNAs in order to be able to repress the corresponding gene expression [34].

Thirteen sets of spacers have highly similar sequences (Table 1). Three sets (set 11, 12 and 13) had minor variations, presumably resulting from random point mutations in some CRISPR alleles rather than independent acquisition events. Interestingly in each case, these mutations were a single nucleotide insertion located at the very end of the spacers. This opens the possibility that the mutated spacer is still able to interfere properly with its target gene. It was previously shown that a phage can escape the CRISPR defense mechanism by as little as 1 bp change in its proto-spacer, but in all examples provided, the mismatch was located at least a few base-pairs inside the spacer [24]. Furthermore, these spacers could be strong evidences for a close link among strains containing them. For example, the a37, a37′ and a37″ spacers are observed adjacent to respectively a6, a5 and a4, respectively. Spacer a37″ is an unusually shorter (29 bp long) spacer, whereas a37′ with an extra GTT at one end has the usual 32 bp in size. The 31 bp spacer a37 lacks one of the final Ts (Table S2). These observations suggest that the above three spacers are the result of a single acquisition event, with a37′ most likely being the ancestor sequence and the two others secondary variants.

**Table 1:** The position of similar proto-spacers in the *Y. pestis* CO92 genome and their distribution in different plague foci.

Set	Spacer ID	Sequence of corresponding proto-spacers@	Gene ID	Position in CO92 genome
1	a71	TCCAATA**CTGACCGTTTTGCTGTAGGTGGCGG**	YPO2116	2384956‥2384987
	a42	**CTGACCGTTTTGCTGTAGGTGGCGG** TGTAGGG		2384963‥2384994
	b23 (rc)*	CATCCAATA **CTGACCGTTTTGCTGTAGGTGGC**		2384954‥2384985
2	a73	**AAGAACAGAGGTAGATGCAGCCTGATC** GCCAG	YPO2106	2378978‥2379009
	a34(rc) *	ATGCC**AAGAACAGAGGTAGATGCAGCCTGATC**		2378983‥2379014
3	a76	TG**ATCGCCAGTTGCCTGCGTTGCTGTTTTTACC**	YPO2106	2378955‥2378987
	a68(rc) *	**ATCGCCAGTTGCCTGCGTTGCTGTTTTTACC** G		2378954‥2378985
	a68′(rc) *	G**ATCGCCAGTTGCCTGCGTTGCTGTTTTTACC**G		2378954‥2378985
4	a74	GCTCTG**CCAAGCTGCAACAATCGCGGCCAACA**	YPO2106	2379146‥2379177
	a19(rc) *	**CCAAGCTGCAACAATCGCGGCCAACA** TTCCTG		2379140‥2379171
5	a75	**CTGGCGCGAGTACCTTCGTCCATTTCATCA** AT	YPO2108	2380168‥2380199
	a36	ATA**CTGGCGCGAGTACCTTCGTCCATTTCATCA**		2380170‥2380202
6	a77	**TTCCAGCGTGTATTTGAGTCGGTCACGGATAA**	YPO2108	2380022‥2380053
	a13 (rc) *	CC**TTCCAGCGTGTATTTGAGTCGGTCACGGATAA**		2380022‥2380055
7	a29	ACGGAGAATCAA**TTCCTACGTTTACTCTCTAAC**	YPO3727	4174095‥4174127
	b6	**TTCCTACGTTTACTCTCTAAC** TCGCCACTCCA		4174084‥4174115
8	a46	CATATTAATGGCTAATAAC**GACATTACATTTA**	YPO2106	2377908‥2377939
	b42	**GACATTACATTTA** TCCGGCCTGAACATGGGGC		2377927‥2377958
9	a56	GG**TTTACGCCGCTGCAATGGCTCAACCGTTCC**	YPO2108	2379677‥2379708
	a81(rc) *	**TTTACGCCGCTGCAATGGCTCAACCGTTCC** AA		2379675‥2379706
10	b47	**AAAATCAGAGTCCCAGCCTGACA** GGTGGCTAA	YPO2106	2379272‥2379303
	c6(rc) *	ACGAATTGA**AAAATCAGAGTCCCAGCCTGACA**		2379263‥2379294
11	b4	**TTCTGGATAGGACAAATAGGATGATTGTATCAG**	-	No homologous in the genome
	b4′	**TTCTGGATAGGACAAATAGGATGATTGTATCAG** G		
12	a37	**TCGTCAATGAATTGCGGGACGTTCCGGCGGT**	-	No homologous in the genome
	a37′	**TCGTCAATGAATTGCGGGACGTTCCGGCGGT** T		
	a37″	**TCGTCAATGAATTGCGGGACGTTCCGGCG**		
13	c3	**CTGAAATACAAATAAAATAAATCGTCGAACA** T	-	No homologous in the genome
	c3′	**CTGAAATACAAATAAAATAAATCGTCGAACA**		

* rc = reverse complement sequence of corresponding spacer.

@ : bolded nucleotides indicate identical region between spacers.

The other 10 sets in Table 1 derive from four genes in the *Y. pestis* genome (Table 1). No similar spacers were observed within the same CRISPR allele. The observation of 2 to 3 spacers originating from very closely related genetic fragments indicated that some loci were hot spots for spacers' acquisition. We collected 200 bp flanking region across the homologous sequences of spacers for further analysis, but no conserved sequences or RNA secondary structure could be identified. A more detailed analysis of proto-spacers will be needed to identify target sequences if they exist.

Seven spacers have one nucleotide difference with the corresponding proto-spacer (Table 2), with G (6/7) or T (1/7) at the 3′ terminus. The G might be added during the spacer acquisition process, rather than by replacement mutation after the spacers' insertion into the CRISPR locus, in which case we would expect to observe the two variant spacers in the population, as seen in sets 11, 12, and 13 (Table 1). In only one spacer (a13) among the seven, the G, which is the second nucleotide from the last one, was replaced by C.

**Table 2:** Spacers with single nucleotide mutation*.

ID	Sequence@	Length(bp)	Gene ID
a11	TCAGTCCCGTTATGGTGCTGGTGTTGCCCGTAA***G***	34	YPO2108
p-a11$	TCAGTCCCGTTATGGTGCTGGTGTTGCCCGTAA***A***		
a13	TTATCCGTGACCGACTCAAATACACGCTGGAA***C***G	34	YPO2108
p-a13$	TTATCCGTGACCGACTCAAATACACGCTGGAA***G***G		
a36	ATACTGGCGCGAGTACCTTCGTCCATTTCATC***G***	33	YPO2108
p-a36$	ATACTGGCGCGAGTACCTTCGTCCATTTCATC***A***		
a43	TCGGCGGTTATGCGGATCATCCCGCTTGGGGC***G***	33	YPO2114
p-a43$	TCGGCGGTTATGCGGATCATCCCGCTTGGGGC***T***		
a76	TGATCGCCAGTTGCCTGCGTTGCTGTTTTTAC***G***	33	YPO2106
p-a76$	TGATCGCCAGTTGCCTGCGTTGCTGTTTTTAC***C***		
b8	TTAACGTAGCCAGGGCGTGTGGAACATGCCTA***G***T	34	YPO2101
p-b8$	TTAACGTAGCCAGGGCGTGTGGAACATGCCTA***T***T		
a68′	CGGTAAAAACAGCAACGCAGGCAACTGGCGAT***G***	33	YPO2106
p-a68′ $	CGGTAAAAACAGCAACGCAGGCAACTGGCGAT***C***		

* The sequences of a11, a13 and b8 come from the sequenced strains (Antiqua and Pestoides F respectively, the GenBank accession no. is described in the section of materials and methods), other three spacers sequences are sequenced by double orientation to confirm the single nucleotide mutation.

@ italic nucleotides indicate a mismatch between the spacer and the prophage.

$ “p-” indicated the corresponding proto-spacers.

Twelve proto-spacers lay between two adjacent genes in the *Y. pestis* genome (marked in grey shade, Table S2). This observation suggests that spacers were acquired from DNA rather than transcribed RNA, unless these intergenic regions were part of an operon. The DNA origin was also suggested by the observation in *S. thermophilus* of short consensus sequence in the vicinity of the proto-spacer, reminiscent of a DNA restriction mechanism [23], [24].

### The diversity of spacers arrays in three CRISPR loci of *Y. pestis*

The three CRISPR loci were present in all the tested isolates, and 35 (60%), 16 (28%) and 7 (12%) alleles are observed in YPa, YPb and YPc, respectively (Table S4). For YPa, 17 spacers were observed in only one isolate (called unique spacers), and the number of spacers per locus ranged from 1 to 14 with an average of 12. For YPb, there are five unique spacers, and the allele size range is 2 to 12 with an average number of 8. For YPc, there was only one unique spacer (c3′, with a single nucleotide difference to c3) and a size range of 1 to 5 with an average number of 3. YPa is the most polymorphic CRISPR locus in *Y. pestis*, in agreement with previous results [18], followed by YPb, and YPc.

By combining spacers arrays of the three loci, 49 genotypes were observed from 131 strains studied in this report (Table S1). By comparing the number of all spacers, types of spacers arrays and proportion of unique spacers between *Y. pestis* and *S. thermophilus* [23], [24], a lower diversity of CRISPR loci was observed in the former.

### Geographic distribution of spacers/spacers arrays and CRISPR clusters of *Y. pestis*

**Figure 2:**
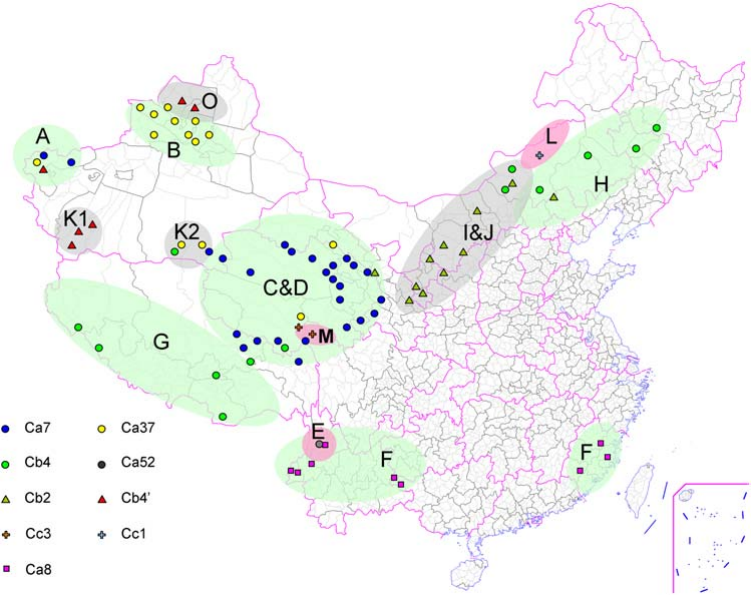
Geographic position of isolates and natural foci on the map of China. Square represents bv. orientalis, triangle bv. mediaevalis, circle bv. antiqua, and cross bv. microtus. If several strains isolated from the same county belonged to the same CRISPR cluster, it was only marked once. Color shadows draw the rough outline of different natural foci, the letters in shadows are focus' names (Table S1).

**Figure 3:**
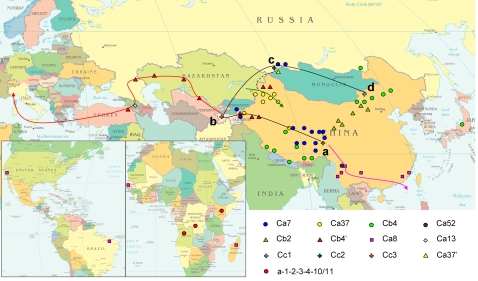
Hypothetic transmission route of *Y. pestis.* Black line shows the main transmission route of *Y. pestis* bv. microtus, red line bv. mediaevalis, pink line bv. orientalis, and green line Ca37. Black dot line shows the potential relationship between Ca37 and Ca37′. Various symbols represent different biovars (see legend of Figure 2 for detail, one more symbol, diamond, is used to represent subsp. *caucasica* in this figure). The spacers array information of isolates from South America and Africa comes from previous research [18]. Red circles represent isolates from Africa, with spacers array “a-1-2-3-4-10/11” in YPa (data from Pourcel *et. al*, 2005). All the known African isolates contain “a10” spacer. Therefore, a10 is possibly a characteristic spacer of isolates from Africa. Nevertheless, we do not consider these isolates as one cluster due to the limited data available from African strains.

In agreement with previous observations, CRISPR loci in *Y. pestis* have conserved spacers in the first part of arrays: “a-1-2-3-4-5-6” in YPa, “b-1-2-3-4” in YPb and “c-1-2-3” in YPc, and these conserved spacers were named as SSSs (Species-Specific Spacers). Most variations in this part can be attributed to random spacer loss, which might be generated by homologous recombination between adjacent DR elements. In contrast, the spacers closer to the leader region are often associated with a specific plague focus or clade (Table S5). They are subsequently called Region-Specific Spacers (RSSs). These spacers are predicted to be acquired more recently, as initially suggested [18] and confirmed by a number of investigations [24], [28], [35]. The geographic distribution of the isolates analyzed in this study is shown on the map of China (Figure 2) and on a world map (Figure 3). Strains with the same spacers array are usually distributed over a specific region. Given the good correlation between spacers arrays and geographical distribution of isolates, all strains studied can be conveniently grouped into 12 clusters, designated by adding a prefix “C” before the name of a representative spacer (most of them are RSS) (Table 3). Using this classification, most natural plague foci have one main cluster except the east side of the Kunlun Mountains (focus K2) and the Pamirs Plateau (focus A) (Table S6).

**Table 3:** Clusters of *Y. pestis* based on CRISPR polymorphism.

Biovar/subspecies	Name of cluster	Spacers arrays*		
		YPa	YPb	YPc
Antiqua	Ca37	a-1-2-3-4-5-6-***37***-?	b-1-2-3-4-10-***23***-?	c-1-2-3-?
	Ca7	a-1-2-3-4-5-6-***7***-?	b-1-2-3-4-?	c-1-2-3
	Ca52	a-1-2-3-5-6-7-***52***-?	b-1-2-3-4	c-1-2-3
	Cb4	a-1-2-3-?	b-1-2-3-4	c-1-2-3
Mediaevalis	Cb2	a-1-2-3-?	b-1-2	c-1-2-3
	Cb4′	a-1-2-3-?	b-1-2-3-4′	c-1-2-3
Orientalis	Ca8	a-1-2-3-4-5-6-7-***8***-?	b-1-2-3-4-***5***-?	c-1-2-3
Microtus (including subsp. *altaica* and *hissarica*)	Cc1	a-1-4-6	b-1-2-3-4	c-1
	Cc2	a-1-4-6	b-1-3-4-10	c-1-2
	Cc3	a-1-4-6	b-1-2-3-4-10	c-1-2-3
Microtus/*caucasica*	Ca13	a-1-2-3-5-***13***-?	b-1-***9***-2-10-11-12	c-1-2-3-5-6
Microtus/*ulegeica*	Ca37′	a-1-2-3-4-5-***37′***-82	b-1-2-3-4-10	c-1-3

* The characteristic spacers of clusters were italic. Question marks represent last part of spacers array, which possibly include RSSs and unique spacers.

Within *Y. pestis*, bv. antiqua strains fall into four clusters (Table 3), Ca37, Ca7, Ca52 and Cb4. The cluster Ca37 possesses the highest number of spacers at YPa (12 spacers per allele on average) and YPb (8 spacers per allele on average) among all the studied strains. Notably, there are 10 proto-spacers in YPO2116 (a gene in a defective bacteriophage) (Figure S1). Their corresponding spacers (a38, a41, a42, a44, a50, a71, b23, b24, b27 and b45) are only observed in Ca37 strains that were mostly isolated from focus B. Based on the hypothetic gene expression regulating function of CRISPR elements, the repression of YPO2116 might have been of importance for *Y. pestis* adaptation to the environment of focus B.

Strains of Cb4 are distributed in both foci G and H, which are far away from each other, with huge variations in environmental and ecological systems. However, strains from these two foci could not be distinguished by biochemical or molecular methods until now [9], [10], [36]. Most strains of the Cb4 (10 of 11, 91%) have spacers array “a1∼3” in YPa, similarly to bv. mediaevalis strains. The b4 spacer in YPb was not present in bv. mediaevalis, hence it could be used to distinguish this antiqua cluster with the two clusters of mediaevalis strains (Table 3). b4′, the characteristic spacer of mediaevalis cluster Cb4′, has only one nucleotide difference with spacer b4 (Table 1), which might indicate a close relationship between Cb4 and Cb4′ strains.

All the strains in the Ca8 cluster belong to bv. orientalis, and two spacers, a8 in YPa and b5 in YPb, were observed in all of them. Half of Ca8 strains have a conserved YPa spacers array “a1∼8”, the other half have a unique spacer added after a8. YPb and YPc in this cluster were invariant, with spacers arrays “b1∼5” and “c1∼3”, respectively. The CRISPR loci of about 150 bv. orientalis strains isolated from other regions of the world have been reported previously [18], and some unique spacers were identified at the end of the spacer array in both YPa and YPb. Our observation is in agreement with the previous results that the spacers array of most bv. orientalis strains were conserved, except that 4 isolates lost the first 4 spacers (a1, a2, a3 and a4) and 1 isolate lost YPa spacer a5 (these 5 strains were isolated from India, South Africa and Vietnam, respectively).

### The atypical strains in central Asia region and China could be assigned to bv. “microtus”

The atypical strains isolated from central Asia region have been named “vole's strains”, which was equivalent to “microtus strains” [2], [3]. Later they were designated as “pestoides” and more recently subdivided into several subspecies, including: *altaica*, *hissarica*, *ulegeica*, *caucasica* and *talassica* [3]. Bv. “microtus” was proposed to depict the isolates from foci L and M of China, which were avirulent to humans and could not ferment arabinose [37]. Because both the “non-*pestis* subspecies” in central Asia region and the microtus strains in China belong to the ancient branches of *Y. pestis* [7] and share common feature of low virulence (or avirulence) to large mammals [3], [37], [38], we suggest to broaden the term “microtus” to the meaning of the term “pestoides” group. According to above definition, the bv. “microtus” should include Cc1 (original bv. microtus strains), Cc2 (subsp. *altaica*), Cc3 (original bv. microtus strains and subsp. *hissarica*), Ca13 (subsp. *caucasica*), Ca37′ (subsp. *ulegeica*), Ca37″ (subsp. *angola*), and subsp. *talassica* (strains were not available for this project). The ability to ferment rhamnose can be used to distinguish the newly defined microtus strains from the other three classical bv. strains [3].

Foci L and M are two distinct *Microtus*-related natural plague foci in China, the phenotypic and biochemical features of the *Y. pestis* isolates from these foci are almost identical, and cannot be distinguished by conventional and genetic methods until now [10], [11]. Here we found that the difference in YPc could be employed as a good marker to distinguish the bv. microtus strains between these two foci. The CRISPR types of strains in foci L and M are Cc1 and Cc3, respectively. Cc3 has two more spacers than Cc1 in YPc. Counting the length of DR sequences, there is a 120 bp difference between the strains from these two loci. It is easy to identify them by PCR-gel electrophoresis method (Figure S2). In order to standardize the nomenclature of bv. microtus, we propose strains from focus L as bv. microtus / *xilingolensis* (N. L. masc. adj. *xilingolensis*, belonging to XiLin Gol Grassland, Inner Mongolia, China, where the strain was isolated) and those from focus M bv. microtus / *qinghaiensis* (N. L. masc. adj. *qinghaiensis*, pertaining to Qinghai, a province of northwest China, where the strain was isolated).

Subsp. *caucasica* strains were originated from Trans-Caucasian-highland foci of the FSU, and showed the same glycerol and nitrate metabolism as the *Y. pestis* bv. antiqua strains [3]. Spacer b9 in YPb of this subsp. is located between two SSSs, b1 and b2. The b9 spacer is also observed in strain C1962002 from the Xinghai County, Qinghai province, China. It seems that C1962002 belongs to the most ancient lineage of *Y. pestis* among all Chinese isolates by DFR analysis [11]. Considering that the subsp. *caucasica* strains are also believed to be ancestral [7], b9 is most probably an ancient (SSS) spacer that was lost in isolates of other clusters.

### The hypothetic evolutionary scenario of *Y. pestis*

**Figure 4:**
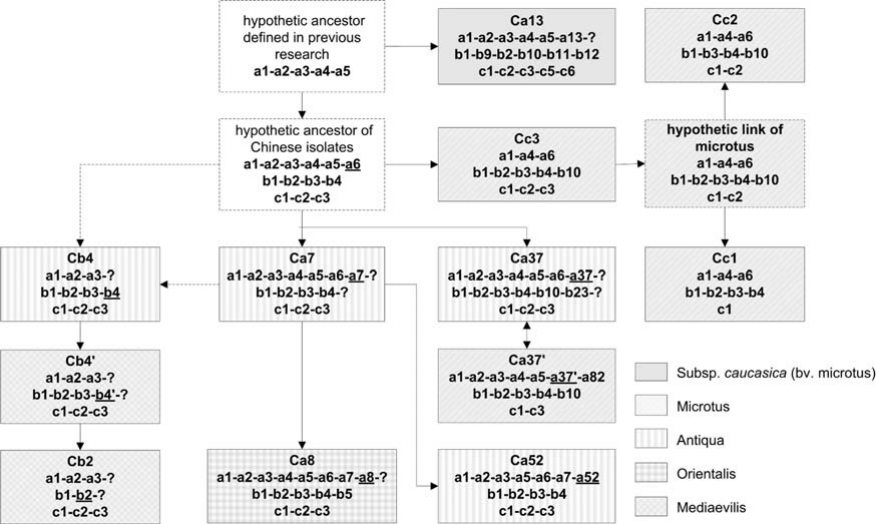
Evolutionary model of *Y. pestis* based on CRISPR polymorphisms. Question marks represent last half part of spacers array. The characteristic spacers are underlined.

The addition of new spacers in a CRISPR locus is polarized towards the leader sequence [18], [35], [39] as initially proposed [18], with few exceptions (the acquisition of a new spacer in an interstitial position concomitantly with the loss of multiple spacers [24]). Because no RSSs or unique spacers were observed in SSSs part of spacers array in this research, we supposed that the latter situation did not occur in *Y. pestis* and assumed that the information preserved in spacers arrays would show the directionally evolutionary record of this species. Here we propose an hypothetic evolutionary model of *Y. pestis* based upon the spacers array of all three CRISPR loci (Figure 4) and according to the general rules of CRISPR evolution [18], [19].

Spacer a6 (SSS) can be predicted to be very ancient, since it had to be acquired before a37, and the very similar spacer a37″ has already been in the ancestral strain “Angola”. Spacer a6 is also present in a number of microtus strains. The most parsimonious hypothesis is that “a6” was lost in the Cb4, Cb4′ and Cb2. The poorly informative “a-1-2-3” allele may descend from multiple ancestors. Nevertheless, in consideration of geographic connection and overlapping of the isolates belonging to the clusters Ca7 and Cb4, Ca7 is a candidate ancestor for Cb4.

### A transmission route of bv. microtus in both China and the FSU area

**Figure 5:**
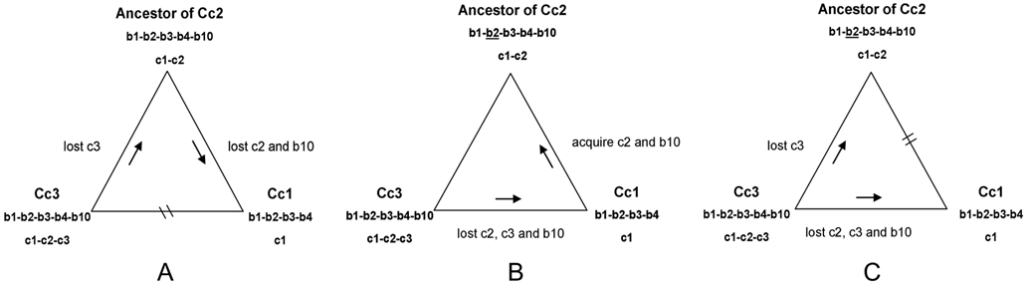
Evolutionary models of bv. microtus Cc3, Cc2 and Cc1 isolates. A: A process from Cc3, through an ancestor of Cc2, to Cc1 with the loss of a series of spacers. B: The model proposes that Cc3 evolved to Cc1 by losing c2, c3 and b10, then to ancestor of Cc2 by acquiring c2 and b10. C: The model proposes that ancestor of Cc2 and Cc1 had no direct relation, but were formed separately by losing different spacers of Cc3.

In this research, the isolates from four different microtus-related plague foci (L, M, 34 and 36) were divided into three clusters (Cc1, Cc2 and Cc3). Because most (90%) spacers arrays in YPc are “c1∼3” and the ancient subsp. *caucasica* isolates had longer array (“c-1-2-3-5-6”), the shorter arrays “c-1-2” and “c-1” in Cc2 and Cc1, respectively, are likely the result of more recent deletion of spacers. Similarly, the spacers array “b-1-2-3-4-10” would generate “b-1-3-4-10” with the loss of b2 spacer. Such process was irreversible because b2 could not insert into the spacers array in its descendants. Therefore, we predict the existence of an ancestor of Cc2 with spacers array “b-1-2-3-4-10” in YPb. Accordingly, evolutionary models among Cc1, Cc3 and ancestor of Cc2 were proposed in Figure 5 for illustrating a tentative evolution scenario. Figure 5-B showed that c2 and b10 as pre-acquired spacers were lost first and then acquired again in the process. However, there are no report of the same two spacers present in one CRISPR locus until now, which seems to say that it is highly unlikely to acquire the same spacer twice. Therefore, this process was unreasonable. Figure 5-C proposes that ancestor of Cc2 and Cc1 had no direct relation. Isolates of Cc2 belong to subsp. *altaica*. This subtype is distributed along the Altai Mountains in Russia and across Mongolia, next to focus L in China, where the Cc1 is located, and no natural barrier is separating Cc1 and Cc2. Furthermore, it seems coincidental that the same spacer c3 was deleted in two different evolutionary directions. Therefore, the process showed in Figure 5-C is unreasonable too. Altogether, the process shown in Figure 5-A is the most likely evolutionary model of bv. microtus strains in these four plague foci.

According to the model above, we marked the possible connections of bv. microtus strains on the map (Figure 3), which moved around the Takla Makan Desert and Jungger Basin. Starting from Tadjikistan (subsp. *hissarica*), one branch (b-a) passed through the Kunlun Mountains, and went to the Qinghai-Tibet Plateau. A second branch (b-c-d) went north via the Pamirs Plateau, the Tianshan Mountains, the Altai Mountains and the Inner Mongolian Plateau. Bv. microtus isolates other than representatives of the most ancestral subsp. *caucasica* may originate from Tadjikistan (mostly between a and c in Figure 3) and expand along this line. According to Achtman *et al.* [7], bv. microtus was one of the oldest lineage of *Y. pestis*, therefore it can be hypothesized that *Y. pestis* emerged along this line. Isolates of Ca7 distributed in focus A, and foci 37 and 41 were on this route (blue spots on Figure 3), which supports such hypothesis. Therefore, this line might also be the main transmission route of *Y. pestis*. Some clones originated from this main route, like Ca8 (bv. orientalis, pink line in Figure 3) and Cb4′ (bv. mediaevalis, red line in Figure 3), have been subsequently transmitted to other places of the world by human business activity, like business through the silk road, or other coincident events, like the Caffa siege in 1346 [40].

Focus B in China extends along the Tianshan Mountain, connecting with Pre-Balkhash (focus 30) of Kazakhstan, and had been proposed to be the point of entry of *Y. pestis* strains into China from Central Asia [10]. The YPa polymorphism would fit with the expected evolutionary direction of *Y. pestis* in this long narrow focus. From west to east, focus B can be separated into three sections of similar size: B4, B3 and B2 (Figure S3). The majority of B4 isolates have the most complete SSSs, with “a-1-**2**-3-4-**5**-6-37-**38**-39” in its first part of spacers array. In contrast, some B3 isolates miss spacer a2, others a5 and a38. The latter subgroup lost a2 to form the spacers array observed in B2 isolates. The internal deletions of spacers indicate that the origin of Ca37 was from west side of the Tianshan Mountain (located on the main transmission route) and the evolutionary direction was from west to east (Figure 3 and Figure S3).

### Conclusion

The research on CRISPR is still in its infant period. The analyses of spacers identified in the present research are helping to provide some rules for further illuminating the mechanism of CASS. The region-specific features made CRISPR loci as robust and easily standardized typing tools for *Y. pestis*, which will be helpful in rapidly tracing the source of outbreaks, as well as setting-up effective prevention and treatment during plague epidemic. Such results open the way to the development of a spoligotyping assay, which can be applied to any new isolate of *Y. pestis*. We proposed an evolutionary model of *Y. pestis* based on polymorphism of CRISPR loci, and our model suggested a main transmission route of *Y. pestis*. The branches derived from this main route may lead to the formation of natural plague foci in other places of the world. Nevertheless, the exact evolutionary scenario still needs to be uncovered by analyzing more isolates.

## Materials and Methods

### Bacterial strains

Three hundred and sixty-six *Y. pestis* strains from China, 35 strains from the FSU, and 4 strains from Mongolia were initially genotyped by MLVA as previously described (Pourcel et al., 2004). The three CRISPR loci were then investigated in a representative collection of 125 Chinese strains plus the FSU and Mongolian strains (Table S1). The strains were collected by the Xinjiang, Yunnan, Qinghai and Inner Mongolia Center for Disease Prevention & Control, China; as well as Antiplague Research Institute of Siberia and Far East, Irkutsk, Russia and the Russian Research Anti-Plague Institute “Microbe”, Saratov, Russia. All the strains were cultivated in the Luria-Bertani broth and the genomic DNAs were extracted using the conventional phenol-chloroform extraction method.

### CRISPR loci amplification

Three CRISPR loci (YPa, YPb, YPc) were amplified respectively with the following primer pairs that targeted the region flanking the CRISPR [18]. YP1-L (5′-AATTTTGCTCCCCA AATAGCAT-3′) and YP1-R (5′-TTTTCCCCATTAGCGAAATAAGT A -3′) were used for amplifying the YPa region; YP2-L (5′-ATATCCTGCTTACCGAGGGT-3′) and YP2-R (5′-AATCAGCCACGCTCT GTCTA-3′) for YPb; and YP3-L (5′-GCCAAGGGATTAGTGAGTTAA-3′) and YP3-R (5′-TTTA CGCATTTTGCGCCATTG-3′) for YPc. The amplifications were performed in a 30 µl volume containing 10 ng of DNA template, 0.5 µM of each primer, 1 unit of Taq DNA polymerase, 200 µM of dNTPs, and 10×PCR buffer containing 500 mM KCl, 0.1 M Tris HCl (pH 8.3) and 25 mM MgCl_2_. The cycling condition were 95°C for 5 min, followed by 30 cycles of denaturation at 95°C for 40 s, annealing at 58°C for 40 s, extension at 72°C for 1 min, and a final extension at 72°C for 5 min in a MJ-Research PTC-225 PCR machine. An aliquot of 5 µL of the product was subjected to electrophoresis on 1.2% agarose gel in 0.5×TBE buffer (0.045 mM Tris-boracic acid, 1 mM EDTA, pH 8.0). The gels were stained with ethidium bromide for visualization under UV light.

### Purification and sequencing

PCR products were purified by using TIAN quick Midi Purification Kit (TIANGEN Biotech Co., China) following the user's manual. The purified products were sequenced on an Applied Biosystems 3730 automated DNA sequencer by the dye termination method. Sequence assembly and editing were performed with the Seqman module of the DNAstar package (DNAstar Inc., Madison, Wis.)

### *In silico* analysis

Seven *Y. pestis* genomes (Antiqua: NC_008150, CO92: NC_003143, KIM: NC_004088, Nepal516: NC_008149, Pestoides F: NC_009381, Microtus str. 91001: NC_005810, Angola: NC_010159) and the BLAST software were downloaded from NCBI website (http://www.ncbi.nlm.nih.gov). The spacer sequences were searched against GenBank using BLAST to find a homologous sequence [41]. The spacers arrays were acquired and analyzed online by using the “CRISPR Finder Tool” and “spacers dictionary” tools in CRISPRs database (http://crispr.u-psud.fr/) [22], [42]–[44]. The RNA secondary structural prediction is performed using the RNAstructure 4.5 software [45].

### Spacer nomenclature and the name of natural plague foci

Because of the large number of new spacers identified in this work, the use of the 26-letter alphabet was not suitable; therefore, a new nomenclature system was employed to designate spacers. The prefix a, b, or c refers to the three CRISPR loci YPa (YP1), YPb (YP2) and YPc (YP3), respectively, and spacers were numbers. As a result, for YP1 spacer “a” used once by previous report will be designated here as “a1”, allele “YP1-abcde” is now “a1-a2-a3-a4-a5”, or in an abbreviated format “a-1-2-3-4-5”, or the more abbreviated “a1∼5” (this format can be used only when the spacers' number are continuous). The start of spacer array is recorded from the opposite side of the leader sequence. The name of natural plague foci of China is “focus+a capital letter”, like “focus B” [9]; natural plague foci of the FSU are named by “focus+a number”, like “focus 34” [3]. The geographic position and background data for the natural plague foci were documented by previous reports [3], [9].

## Supporting Information

Figure S1 The distribution of proto-spacers in prophage genes
(0.78 MB TIF)

Figure S2 Gel electrophoresis results of PCR products of isolates from focus L (Cc1) and M (Cc3). The ladder of marker was 2000, 1000, 750, 500, 250, 100 from top to bottom. From left to right, the first ten strands in fist line were products of isolates from focus L, the others 30 strands were products of isolates from focus M.
(4.22 MB TIF)

Figure S3 Evolutionary models of isolates from focus B. A: Evolutionary models. The number in bracket is the amount of isolates from corresponding region. “?” represent some RSSs and unique spacers. B: The geography position of focus B.
(1.91 MB TIF)

Table S1 Properties of strains
(0.05 MB XLS)

Table S2 Spacers dictionary
(0.05 MB XLS)

Table S3 Diversity of spacer sequence
(0.03 MB DOC)

Table S4 Diversity of spacer's array
(0.03 MB DOC)

Table S5 Distribution of spacers
(0.05 MB XLS)

Table S6 Distribution of CRISPR clusters
(0.11 MB DOC)

## References

[1] Perry RD, Fetherston JD (1997). *Yersinia pestis*–etiologic agent of plague.. Clin Microbiol Rev.

[2] Gage KL, Kosoy MY (2005). Natural history of plague: perspectives from more than a century of research.. Annu Rev Entomol.

[3] Anisimov AP, Lindler LE, Pier GB (2004). Intraspecific diversity of *Yersinia pestis*.. Clin Microbiol Rev.

[4] Greenfield RA, Drevets DA, Machado LJ, Voskuhl GW, Cornea P (2002). Bacterial pathogens as biological weapons and agents of bioterrorism.. Am J Med Sci.

[5] Grygorczuk S, Hermanowska-Szpakowicz T (2002). [*Yersinia pestis* as a dangerous biological weapon].. Med Pr.

[6] Inglesby TV, Dennis DT, Henderson DA, Bartlett JG, Ascher MS (2000). Plague as a biological weapon: medical and public health management. Working Group on Civilian Biodefense.. Jama.

[7] Achtman M, Morelli G, Zhu P, Wirth T, Diehl I (2004). Microevolution and history of the plague bacillus, *Yersinia pestis*.. Proc Natl Acad Sci U S A.

[8] Pourcel C, Andre-Mazeaud F, Neubauer H, Ramisse F, Vergnaud G (2004). Tandem repeats analysis for the high resolution phylogenetic analysis of *Yersinia pestis*.. BMC Microbiol.

[9] Zhou D, Han Y, Song Y, Huang P, Yang R (2004). Comparative and evolutionary genomics of *Yersinia pestis*.. Microbes Infect.

[10] Zhou D, Han Y, Song Y, Tong Z, Wang J (2004). DNA microarray analysis of genome dynamics in *Yersinia pestis*: insights into bacterial genome microevolution and niche adaptation.. J Bacteriol.

[11] Li Y, Dai E, Cui Y, Li M, Zhang Y (2008). Different region analysis for genotyping *Yersinia pestis* isolates from China.. PLoS ONE.

[12] Hai R, Yu DZ, Wei JC, Xia LX, Shi XM (2004). [Molecular biological characteristics and genetic significance of *Yersinia pestis* in China].. Zhonghua Liu Xing Bing Xue Za Zhi.

[13] Achtman M, Zurth K, Morelli G, Torrea G, Guiyoule A (1999). *Yersinia pestis*, the cause of plague, is a recently emerged clone of *Yersinia pseudotuberculosis*.. Proc Natl Acad Sci U S A.

[14] Guiyoule A, Grimont F, Iteman I, Grimont PA, Lefevre M (1994). Plague pandemics investigated by ribotyping of *Yersinia pestis* strains.. J Clin Microbiol.

[15] Lucier TS, Brubaker RR (1992). Determination of genome size, macrorestriction pattern polymorphism, and nonpigmentation-specific deletion in *Yersinia pestis* by pulsed-field gel electrophoresis.. J Bacteriol.

[16] Lindstedt B-A (2005). Multiple-locus variable number tandem repeats analysis for genetic fingerprinting of pathogenic bacteria.. Electrophoresis.

[17] Klevytska AM, Price LB, Schupp JM, Worsham PL, Wong J (2001). Identification and characterization of variable-number tandem repeats in the *Yersinia pestis* genome.. J Clin Microbiol.

[18] Pourcel C, Salvignol G, Vergnaud G (2005). CRISPR elements in *Yersinia pestis* acquire new repeats by preferential uptake of bacteriophage DNA, and provide additional tools for evolutionary studies.. Microbiology.

[19] Vergnaud G, Li Y, Gorge O, Cui Y, Song Y (2007). Analysis of the three *Yersinia pestis* CRISPR loci provides new tools for phylogenetic studies and possibly for the investigation of ancient DNA.. Adv Exp Med Biol.

[20] Mojica FJ, Diez-Villasenor C, Soria E, Juez G (2000). Biological significance of a family of regularly spaced repeats in the genomes of Archaea, Bacteria and mitochondria.. Mol Microbiol.

[21] Jansen R, Embden JD, Gaastra W, Schouls LM (2002). Identification of genes that are associated with DNA repeats in prokaryotes.. Mol Microbiol.

[22] Grissa I, Vergnaud G, Pourcel C (2007). The CRISPRdb database and tools to display CRISPRs and to generate dictionaries of spacers and repeats.. BMC Bioinformatics.

[23] Horvath P, Romero DA, Coute-Monvoisin AC, Richards M, Deveau H (2007). Diversity, activity and evolution of CRISPR loci in *Streptococcus thermophilus*.. J Bacteriol.

[24] Deveau H, Barrangou R, Garneau JE, Labonte J, Fremaux C (2007). Phage response to CRISPR-encoded resistance in *Streptococcus thermophilus*.. J Bacteriol.

[25] Barrangou R, Fremaux C, Deveau H, Richards M, Boyaval P (2007). CRISPR provides acquired resistance against viruses in prokaryotes.. Science.

[26] Bolotin A, Quinquis B, Sorokin A, Ehrlich SD (2005). Clustered regularly interspaced short palindrome repeats (CRISPRs) have spacers of extrachromosomal origin.. Microbiology.

[27] Mojica FJ, Diez-Villasenor C, Garcia-Martinez J, Soria E (2005). Intervening sequences of regularly spaced prokaryotic repeats derive from foreign genetic elements.. J Mol Evol.

[28] Lillestol RK, Redder P, Garrett RA, Brugger K (2006). A putative viral defence mechanism in archaeal cells.. Archaea.

[29] Goyal M, Saunders NA, van Embden JD, Young DB, Shaw RJ (1997). Differentiation of *Mycobacterium tuberculosis* isolates by spoligotyping and IS6110 restriction fragment length polymorphism.. J Clin Microbiol.

[30] Brudey K, Driscoll JR, Rigouts L, Prodinger WM, Gori A (2006). *Mycobacterium tuberculosis* complex genetic diversity: mining the fourth international spoligotyping database (SpolDB4) for classification, population genetics and epidemiology.. BMC Microbiol.

[31] Mokrousov I, Limeschenko E, Vyazovaya A, Narvskaya O (2007). *Corynebacterium diphtheriae* spoligotyping based on combined use of two CRISPR loci.. Biotechnol J.

[32] Haft DH, Selengut J, Mongodin EF, Nelson KE (2005). A guild of 45 CRISPR-associated (Cas) protein families and multiple CRISPR/Cas subtypes exist in prokaryotic genomes.. PLoS Comput Biol.

[33] Derbise A, Chenal-Francisque V, Pouillot F, Fayolle C, Prevost MC (2007). A horizontally acquired filamentous phage contributes to the pathogenicity of the plague bacillus.. Mol Microbiol.

[34] Sorek R, Kunin V, Hugenholtz P (2007). CRISPR-a widespread system that provides acquired resistance against phages in bacteria and archaea.. Nat Rev Microbiol.

[35] Tyson K, Metheringham R, Griffiths L, Cole J (1997). Characterisation of *Escherichia coli* K-12 mutants defective in formate-dependent nitrite reduction: essential roles for *hem*N and the *men*FDBCE operon.. Arch Microbiol.

[36] Dai E, Tong Z, Wang X, Li M, Cui B (2005). Identification of different regions among strains of *Yersinia pestis* by suppression subtractive hybridization.. Res Microbiol.

[37] Zhou D, Tong Z, Song Y, Han Y, Pei D (2004). Genetics of metabolic variations between *Yersinia pestis* biovars and the proposal of a new biovar, microtus.. J Bacteriol.

[38] Song Y, Tong Z, Wang J, Wang L, Guo Z (2004). Complete genome sequence of *Yersinia pestis* strain 91001, an isolate avirulent to humans.. DNA Res.

[39] Barrangou R, Yoon SS, Breidt F, Fleming HP, Klaenhammer TR (2002). Identification and characterization of *Leuconostoc fallax* strains isolated from an industrial sauerkraut fermentation.. Appl Environ Microbiol.

[40] Wheelis M (2002). Biological warfare at the 1346 siege of Caffa.. Emerg Infect Dis.

[41] Altschul SF, Gish W, Miller W, Myers EW, Lipman DJ (1990). Basic local alignment search tool.. J Mol Biol.

[42] Grissa I, Bouchon P, Pourcel C, Vergnaud G (2007). On-line resources for bacterial micro-evolution studies using MLVA or CRISPR typing.. Biochimie.

[43] Grissa I, Vergnaud G, Pourcel C (2007). CRISPRFinder: a web tool to identify clustered regularly interspaced short palindromic repeats.. Nucleic Acids Res.

[44] Grissa I, Vergnaud G, Pourcel C (2008). CRISPRcompar: a website to compare clustered regularly interspaced short palindromic repeats.. Nucleic Acids Res.

[45] Mathews DH, Disney MD, Childs JL, Schroeder SJ, Zuker M (2004). Incorporating chemical modification constraints into a dynamic programming algorithm for prediction of RNA secondary structure.. Proc Natl Acad Sci U S A.

