# Supplementary figures and images for: Insight into Microevolution of Yersinia pestis by Clustered Regularly Interspaced Short Palindromic Repeats
Source: PLoS One. 2008 Jul 9;3(7):e2652. doi: 10.1371/journal.pone.0002652 (PMC2440536; doi:10.1371/journal.pone.0002652)

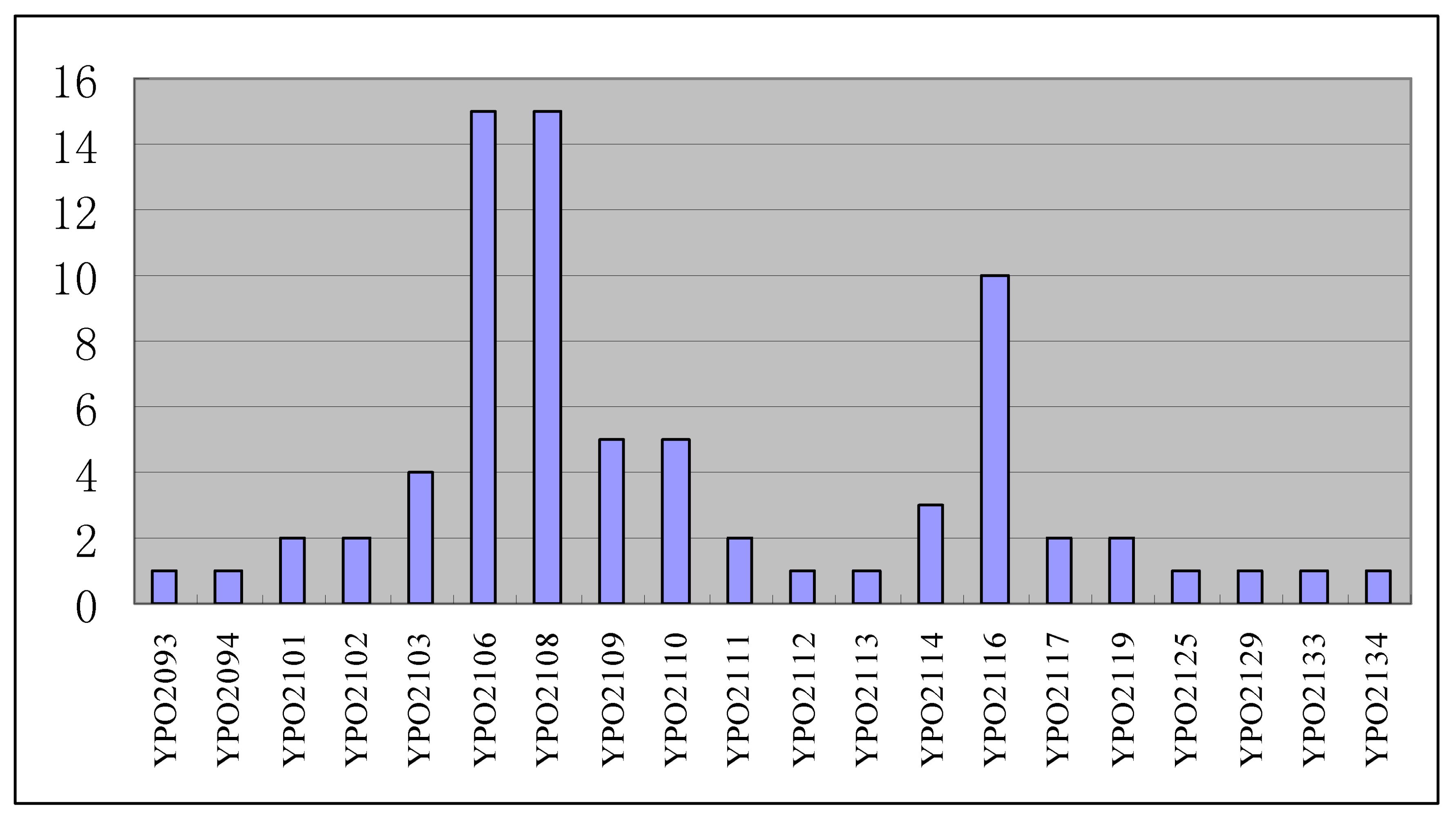

Supplement: Figure S1 — The distribution of proto-spacers in prophage genes (0.78 MB TIF) [file pone.0002652.s001.tif]

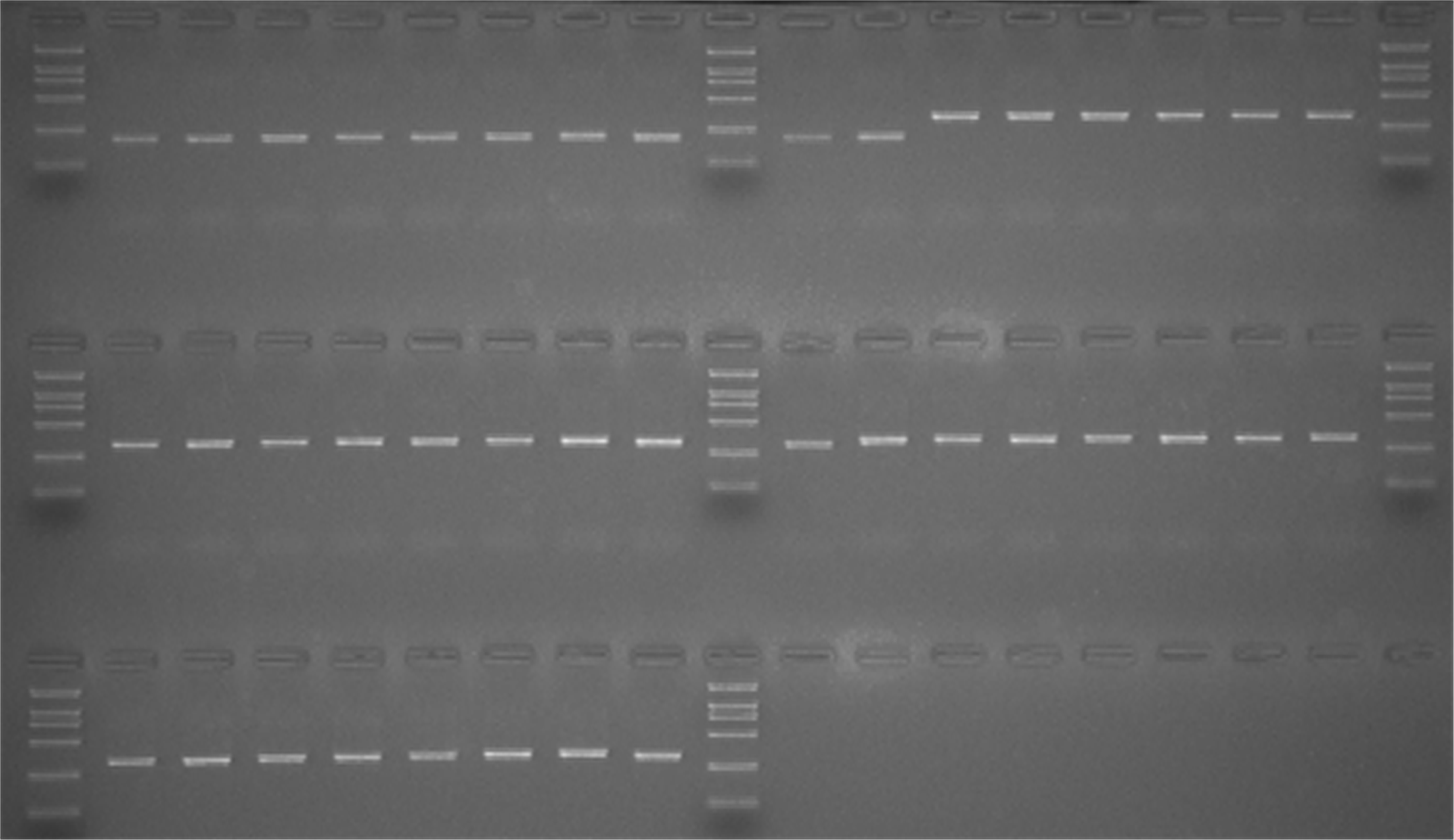

Supplement: Figure S2 — Gel electrophoresis results of PCR products of isolates from focus L (Cc1) and M (Cc3). The ladder of marker was 2000, 1000, 750, 500, 250, 100 from top to bottom. From left to right, the first ten strands in fist line were products of isolates from focus L, the others 30 strands were products of isolates from focus M. (4.22 MB TIF) [file pone.0002652.s002.tif]

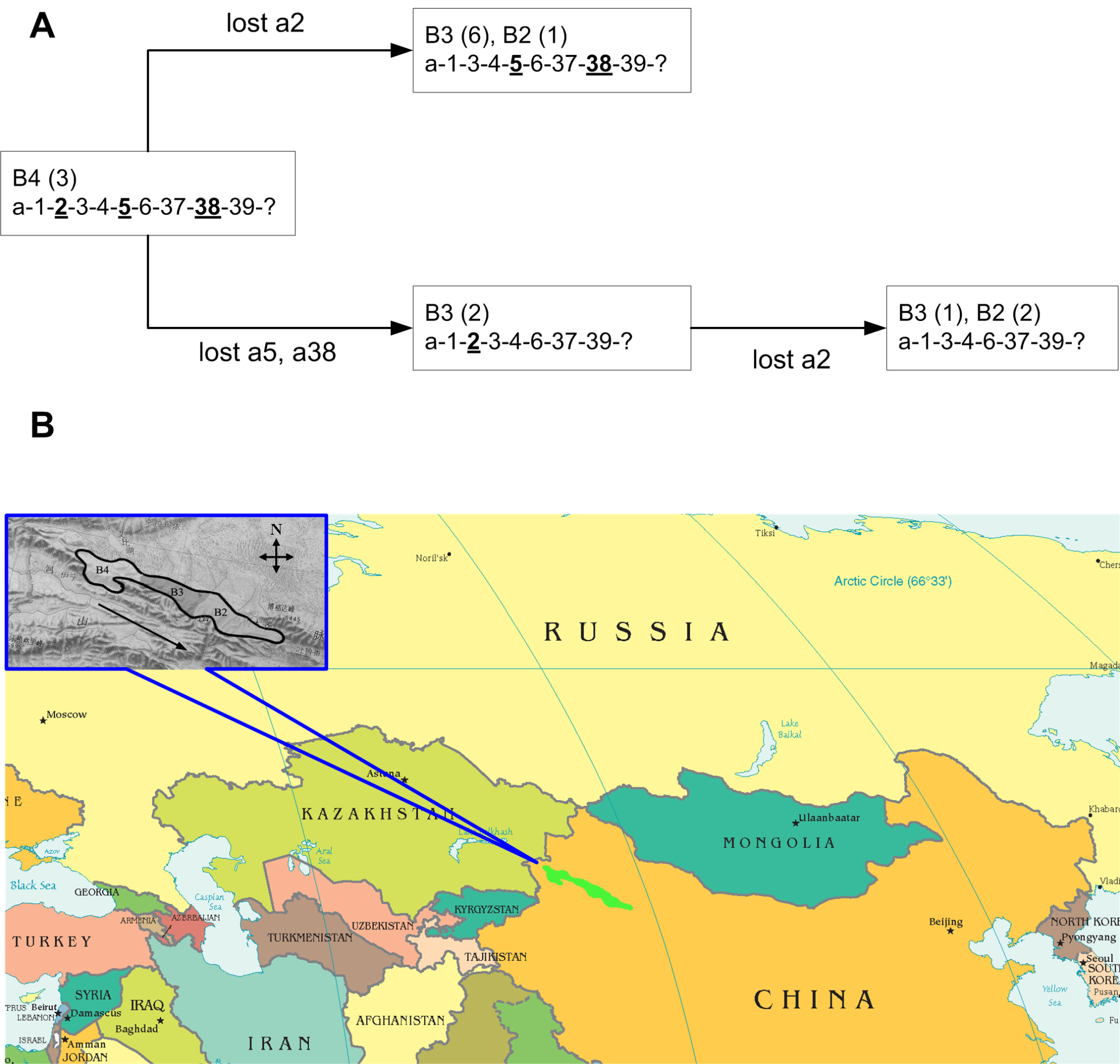

Supplement: Figure S3 — Evolutionary models of isolates from focus B. A: Evolutionary models. The number in bracket is the amount of isolates from corresponding region. “?” represent some RSSs and unique spacers. B: The geography position of focus B. (1.91 MB TIF) [file pone.0002652.s003.tif]
