# Supplementary material for: Insight into Microevolution of Yersinia pestis by Clustered Regularly Interspaced Short Palindromic Repeats
Source: PLoS One. 2008 Jul 9;3(7):e2652. doi: 10.1371/journal.pone.0002652 (PMC2440536; doi:10.1371/journal.pone.0002652)
Supplement: Table S3 — Diversity of spacer sequence (0.03 MB DOC) [file pone.0002652.s006.doc]

**Supplementary Table S3. Diversity of spacer sequence**

|  | **YPa** | **YPb** | **YPc** | **Total** | **Percent content** |
| --- | --- | --- | --- | --- | --- |
| number of spacers | 83 | 37 | 11 | 131 |  |
| without proto-spacer | 14 | 13 | 5 | 32 | 24% |
| with proto-spacer on non-prophage region of genome | 18 | 3 | 1 | 22 | 17% |
| with proto-spacer on prophage region of genome | 51 | 21 | 5 | 77 | 59% |
