# Supplementary material for: Insight into Microevolution of Yersinia pestis by Clustered Regularly Interspaced Short Palindromic Repeats
Source: PLoS One. 2008 Jul 9;3(7):e2652. doi: 10.1371/journal.pone.0002652 (PMC2440536; doi:10.1371/journal.pone.0002652)
Supplement: Table S4 — Diversity of spacer's array (0.03 MB DOC) [file pone.0002652.s007.doc]

**Supplementary Table S4. Diversity of spacer’**s array

|  | **YPa** | **YPb** | **YPc** |
| --- | --- | --- | --- |
| Average number of spacers per locus | 12 | 8 | 3 |
| Minimum number of spacers per locus | 1 | 2 | 1 |
| Maximum number of spacers per locus | 14 | 12 | 5 |
| Number of unique array | 35 | 16 | 7 |
| Unique spacers | 17 | 5 | 1 |
