# Supplementary material for: Insight into Microevolution of Yersinia pestis by Clustered Regularly Interspaced Short Palindromic Repeats
Source: PLoS One. 2008 Jul 9;3(7):e2652. doi: 10.1371/journal.pone.0002652 (PMC2440536; doi:10.1371/journal.pone.0002652)
Supplement: Table S6 — Distribution of CRISPR clusters (0.11 MB DOC) [file pone.0002652.s009.doc]

**Supplementary Table S6. Distribution of** CRISPR clusters

| **Focus or Subfocus** | | **Strain amounts** | **Clusters based on CRISPR polymorphism*** | | | | | | | | | | | |
| --- | --- | --- | --- | --- | --- | --- | --- | --- | --- | --- | --- | --- | --- | --- |
|  |  |  | **Ca37** | **Ca7** | **Ca52** | **Cb4** | **Cb2** | **Cb4’** | **Ca8** | **Cc1** | **Cc2** | **Cc3** | **Ca13** | **Ca37’** |
| A | | 4 | 1 | 2 | 0 | 0 | 0 | 1 | 0 | 0 | 0 | 0 | 0 | 0 |
| B | B1 | 2 | 0 | ***2*** | 0 | 0 | 0 | 0 | 0 | 0 | 0 | 0 | 0 | 0 |
|  | B2 | 3 | ***3*** | 0 | 0 | 0 | 0 | 0 | 0 | 0 | 0 | 0 | 0 | 0 |
|  | B3 | 9 | ***9*** | 0 | 0 | 0 | 0 | 0 | 0 | 0 | 0 | 0 | 0 | 0 |
|  | B4 | 4 | ***4*** | 0 | 0 | 0 | 0 | 0 | 0 | 0 | 0 | 0 | 0 | 0 |
| C | | 22 | 0 | ***19*** | 0 | 3 | 0 | 0 | 0 | 0 | 0 | 0 | 0 | 0 |
| D | | 7 | 1 | ***5*** | 0 | 0 | 1 | 0 | 0 | 0 | 0 | 0 | 0 | 0 |
| E | | 4 | 0 | 0 | ***4*** | 0 | 0 | 0 | 0 | 0 | 0 | 0 | 0 | 0 |
| F | | 11 | 0 | 0 | 0 | 0 | 0 | 0 | ***11*** | 0 | 0 | 0 | 0 | 0 |
| G | | 4 | 0 | 0 | 0 | ***4*** | 0 | 0 | 0 | 0 | 0 | 0 | 0 | 0 |
| H | | 5 | 0 | 0 | 0 | ***4*** | 1 | 0 | 0 | 0 | 0 | 0 | 0 | 0 |
| I | | 6 | 0 | 0 | 0 | 0 | ***6*** | 0 | 0 | 0 | 0 | 0 | 0 | 0 |
| J | | 4 | 0 | 0 | 0 | 0 | ***4*** | 0 | 0 | 0 | 0 | 0 | 0 | 0 |
| K | K1 | 6 | 0 | 0 | 0 | 0 | 0 | ***6*** | 0 | 0 | 0 | 0 | 0 | 0 |
|  | K2 | 5 | 2 | 2 | 0 | 0 | 0 | 1 | 0 | 0 | 0 | 0 | 0 | 0 |
| L | | 2 | 0 | 0 | 0 | 0 | 0 | 0 | 0 | ***2*** | 0 | 0 | 0 | 0 |
| M | | 4 | 0 | 0 | 0 | 0 | 0 | 0 | 0 | 0 | ***4*** | 0 | 0 | 0 |
| O | | 3 | 0 | 0 | 0 | 0 | 0 | ***3*** | 0 | 0 | 0 | 0 | 0 | 0 |
| 6 | | 1 | 0 | 0 | 0 | 0 | 0 | 0 | 0 | 0 | 0 | 0 | 1 | 0 |
| 16 | | 1 | 0 | 0 | 0 | 0 | 0 | ***1*** | 0 | 0 | 0 | 0 | 0 | 0 |
| 18 | | 1 | 0 | 0 | 0 | 0 | 0 | ***1*** | 0 | 0 | 0 | 0 | 0 | 0 |
| 21 | | 1 | 0 | 0 | 0 | 0 | 0 | ***1*** | 0 | 0 | 0 | 0 | 0 | 0 |
| 27 | | 1 | 0 | 0 | 0 | 0 | 0 | ***1*** | 0 | 0 | 0 | 0 | 0 | 0 |
| 33 | | 1 | 0 | ***1*** | 0 | 0 | 0 | 0 | 0 | 0 | 0 | 0 | 0 | 0 |
| 34 | | 1 | 0 | 0 | 0 | 0 | 0 | 0 | 0 | 0 | 0 | ***1*** | 0 | 0 |
| 36 | | 1 | 0 | 0 | 0 | 0 | 0 | 0 | 0 | 0 | ***1*** | 0 | 0 | 0 |
| 37 | | 3 | 0 | ***3*** | 0 | 0 | 0 | 0 | 0 | 0 | 0 | 0 | 0 | 0 |
| 38 | | 2 | 0 | 0 | 0 | ***2*** | 0 | 0 | 0 | 0 | 0 | 0 | 0 | 0 |
| 41 | | 1 | 0 | ***1*** | 0 | 0 | 0 | 0 | 0 | 0 | 0 | 0 | 0 | 0 |
| 4 or 5 or 6 | | 2 | 0 | 0 | 0 | 0 | 0 | 0 | 0 | 0 | 0 | 0 | ***2*** | 0 |
| Bayanölgie province, Mongolia | | 3 | 0 | 0 | 0 | 0 | 0 | 0 | 0 | 0 | 0 | 0 | 0 | ***3*** |
| Hentiy province, Mongolia | | 1 | 0 | 0 | 0 | 0 | ***1*** | 0 | 0 | 0 | 0 | 0 | 0 | 0 |
| Sum | | 125 | 20 | 35 | 4 | 13 | 13 | 15 | 11 | 2 | 5 | 1 | 3 | 3 |

*italic numbers represent the main cluster in the corresponding focus.
